# A Framework for Time-Explicit Life Cycle Optimization

**DOI:** 10.1021/acs.est.6c06868

**Published:** 2026-10-28

**Authors:** Timo Diepers, Jan Tautorus, Jan Marcus Hartmann, Niklas von der Assen

**Affiliations:** † Institute of Technical Thermodynamics, RWTH Aachen University, Aachen 52062, Germany; ‡ Interdisciplinary Transformation University Austria, Linz 4040, Austria

**Keywords:** temporal distribution, evolution, transformation pathways, transition, dynamic, prospective life cycle assessment, industrial decarbonization

## Abstract

Life cycle optimization (LCO) selects optimal processes and supply chains from a set of alternatives given environmental constraints and objectives, supporting the design of sustainable systems and their transitions. However, current approaches rely on static assumptions, overlooking two interacting temporal dimensions: *temporal distribution* (flows are spread over time) and *temporal evolution* (processes and supply chains change over time). Because distribution and evolution jointly dictate the magnitude of a flow at a given time, both must be considered simultaneously to accurately optimize against cumulative environmental impacts and time-specific environmental constraints. We present a framework for time-explicit LCO that captures these interdependencies to determine optimal transition pathways. The framework tracks flow timing relative to process installation and dynamically determines flow magnitudes from time-specific process parameters and background supply chains. A case study on integrated methanol and pig iron production demonstrates how time-explicit LCO enables optimization against time-resolved environmental impacts and constraints, revealing mitigation strategies inaccessible to static approaches: prioritizing early high-efficiency technology installation to idle older processes as operational savings outweigh embodied impacts of stranded assets, and avoiding transient resource bottlenecks through diversification. To facilitate widespread application, we provide the open-source Python package optimex, enabling practitioners to design transition pathways that rigorously respect environmental limits.

## Graphical abstract

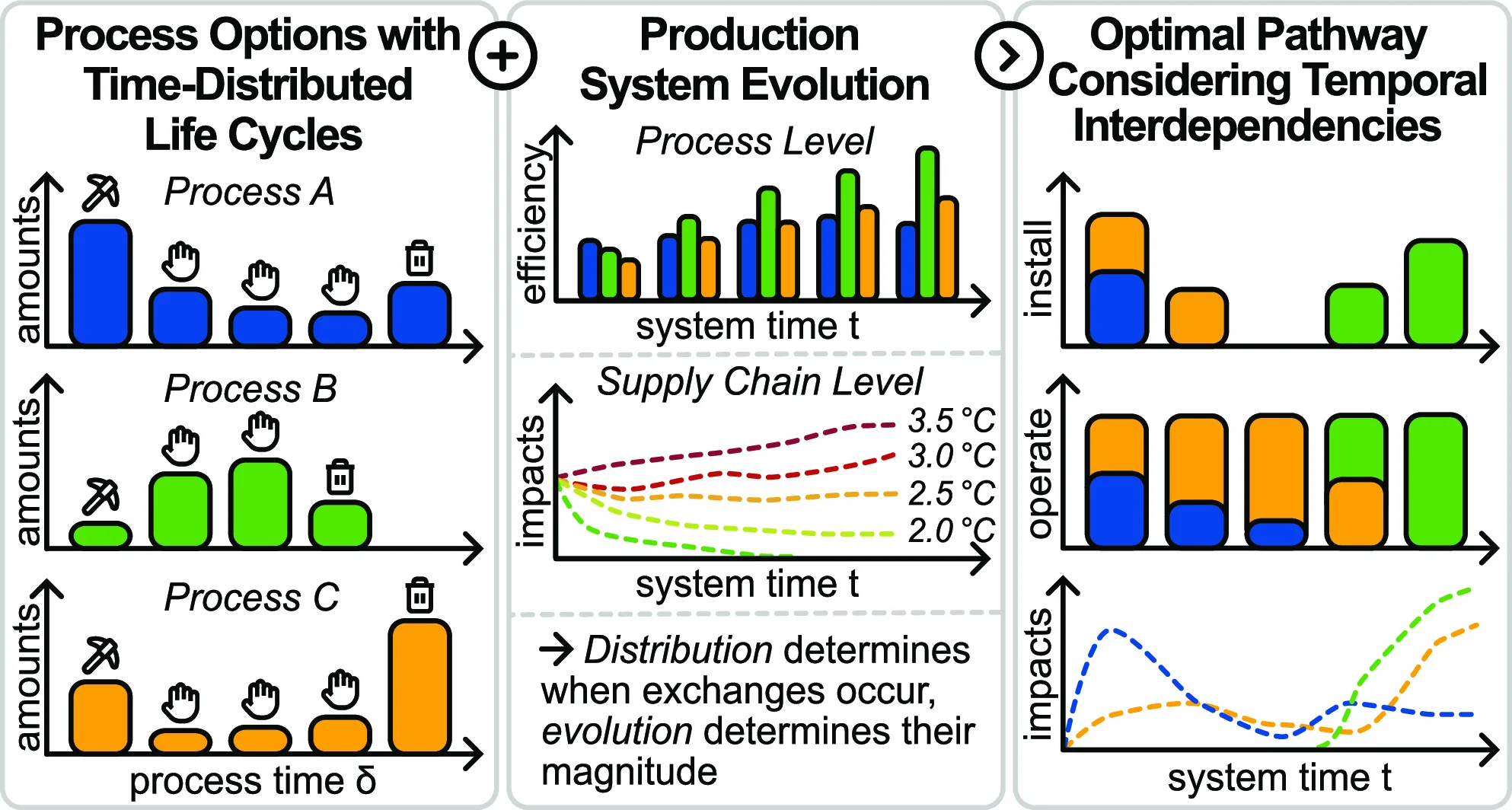

## 1 Introduction

Anthropogenic systems must transition towards environmental sustainability.^1,2^ Environmental sustainability encompasses two fundamental sets of constraints: stock-related limits, such as finite mineral resources^3^ and cumulative carbon budgets,^4,5^ and flow-related limits, dictating maximum rates at which resources can be extracted or emissions assimilated by the biosphere.^3,6^ Any viable transition pathway needs to rigorously respect both sets of constraints.

While integrated assessment models (IAMs) are primary instruments for navigating these transitions, their simplified representations of specific technologies prevent them from identifying the specific bottlenecks within technologies’ supply chains or emission profiles that violate stock- and flow-related environmental limits.^7–9^ Sector- or process-specific optimization models improve on the technological resolution, yet they often neglect upstream supply chains or environmental impact categories besides climate change, risking environmental burden shifting.^10,11^

To holistically consider a broad range of environmental impact categories across entire supply chains, researchers increasingly couple sector- or process-specific optimization models with life cycle assessment (LCA) in life cycle optimization (LCO).^11–17^ Traditional LCA models, and the LCO models built on them, are static, assuming all intermediate flows (i.e., exchanges within the production system) and elementary flows (i.e., exchanges between production system and environment) occur simultaneously. This assumption neglects the temporal interdependencies between two fundamental temporal dimensions: *temporal distribution* and *temporal evolution*.^18^ Temporal distribution defines *when* processes and their intermediate and elementary flows occur, as real-world life cycles can span years or decades and their flows are spread out accordingly.^19–22^ Temporal evolution defines *what* the state of the product system and its environment is at a given time, as processes, supply chains and environmental conditions change over time.^23–26^

For transition pathways, these two temporal dimensions are inextricably linked. While *distribution* places a flow at a specific point in time, *evolution* determines the magnitude of a flow depending on the conditions at that specific time. Consequently, both dimensions jointly dictate whether a transition pathway stays within (1) flow-related limits (e.g., annual water consumption), because the timing and the magnitude of a flow together determine when its peak occurs and how high that peak is, and (2) stock-related limits (e.g., accumulated CO_2_ in the atmosphere), because these flows accumulate over the transition period, so that the stock at each point in time follows from the stock reached previously plus the flows occurring within the current period. LCO models therefore need to account for these temporal interdependencies to ensure environmentally sustainable pathways. In other words, LCO models must be *time-explicit*, defined by Müller et al.^18^ as “simultaneously account­[ing] for temporal distribution and temporal evolution [···] considering the timing of processes and emissions as well as the state of technologies and supply chains at the respective points in time”.

The time-explicit *assessment* of transition pathways is enabled by time-explicit LCA.^18^ However, time-explicit LCA can only assess a single predefined pathway, and not *design* a pathway that is optimal with respect to an objective, such as minimum climate change impacts. As a first step toward designing optimal transitions, time-explicit assessments of predefined pathways have been combined with an iterative adjustment of these pathways toward a normative target,^27^ or with optimization over a limited set of design parameters through surrogate models of the life cycle inventory.^28,29^ These approaches, however, remain confined to a set of pathways that is fixed before the assessment, and thus risk overlooking better alternatives. Accounting for temporal interdependencies makes overlooking better alternatives particularly likely, as the interactions between installation timing, flow timing, and evolving supply chains are complex and therefore challenging to anticipate. The direct design of optimal pathways therefore requires the full integration of time-explicit life cycle impacts into an LCO model.

Existing attempts to introduce time into LCO focus on multi-period optimization models, which resolve time at the level of the decision: they split the planning horizon into periods and choose in which period a process is installed and operated. Such models range from early approaches that indirectly use LCA results in multi-period optimization models, such as Hugo and Pistikopoulos,^30^ to the full integration of multi-period optimization into the matrix-based LCA framework^31^ by Zibunas et al.;^32^ a detailed overview of this literature is provided by Hülagü et al.^33^ In these multi-period models, the life cycle behind each decision stays static, meaning that every decision draws on a single life cycle inventory that collapses all flows associated with a process and its further supply chains into the period of the decision. In such a model, a process installed in 2040 consumes its construction materials, its operational inputs, and its end-of-life treatment all in 2040, although construction could precede operation by years and decommissioning follows it by decades. Existing multi-period LCO approaches therefore resolve the distribution of decisions across the horizon, but not the temporal distribution of the flows within the life cycle behind each decision. Moreover, as the same inventory is used regardless of when a decision is taken, the temporal evolution of the process and its upstream supply chains remains unresolved as well.

To resolve temporal evolution in multi-period LCO, several approaches have included time-specific inventory data (i.e., reflecting temporal evolution at different time points) instead of a static inventory: Reinert et al.^34^ and Charalambous et al.^35^ couple multi-period energy system optimization models with prospective life cycle inventories, and Thakker and Bakshi^36^ optimize a transition of the chemical industry incorporating learning curves and ongoing decarbonization. Similarly, Hung et al.^37^ consider the temporal evolution of the electricity sector, alongside stock dynamics. However, these contributions likewise disregard the temporal distribution of the life cycles behind the decisions, assuming all flows occur at the moment of a process’s installation or operation.

To our knowledge, the only approach that resolves the life cycle distribution of flows in addition to their evolution is proposed by Hülagü et al.^33^ They place every flow directly at an absolute point in time, capturing time-specific inventory data in time-indexed technosphere and biosphere matrices, with product flows temporally linked through an additional transition matrix. This formulation, however, is only feasible because their model accounts for a single process installation decision at the beginning of the planning horizon, so that only the operation of the installed processes varies over time. For example, the formulation by Hülagü et al.^33^ can optimize which and how many electrolyzers to install in 2020 to satisfy a hydrogen demand from 2020 to 2030, accounting for the progressively decarbonizing electricity these electrolyzers consume.

In contrast, a transition pathway consists of a sequence of installation and operation decisions that the model needs to track through time. A single installation decision at the beginning of the planning horizon does not suffice because (1) transitions span decades, over which existing infrastructure reaches its end of life and has to be replaced, or processes with short lifetimes have to be rebuilt, and (2) when to install is itself a decision variable of a transition. For example, installing later could benefit from a more efficient process, while installing earlier could displace an emission-intensive process sooner. An optimization model can only find optimal trade-offs if installation times remain decision variables throughout the planning horizon.

Overall, optimizing a transition pathway requires a model that tracks the intermediate and elementary flows of a sequence of installation and operation decisions over a planning horizon, evaluating every flow with the process and supply chain conditions at the point in time at which the flow occurs. Only these temporally resolved elementary flows reveal when emissions and resource extractions occur and how large they are at that time, and thus whether a pathway stays within stock- and flow-related environmental limits.

In this work, we propose a framework for time-explicit LCO of transition pathways. By simultaneously considering the temporal distribution and evolution of process’s intermediate and elementary flows, our framework identifies pathways that rigorously respect both stock- and flow-related environmental constraints. To achieve this, our framework tracks the timing of flows relative to a process’s installation, and dynamically determines the magnitudes of flows at that specific time from time-specific process parameters and background supply chains. To facilitate the widespread adoption of time-explicit LCO, we provide an implementation of the framework in the open-source Python package optimex, which integrates with the Brightway LCA framework^38^ and the pyomo optimization framework.^39^ The application of time-explicit LCO is demonstrated in a case study on the transition of an integrated industrial system co-producing methanol and pig iron.

## 2 Time-Explicit Life Cycle Optimization Framework

As the basis for time-explicit LCO, we first review the mathematical formulation of static LCO in Section 2.1. Building on this, we then introduce a mathematical framework for time-explicit LCO in Section 2.2, extending static LCO to simultaneously consider temporal distribution and evolution of processes’ intermediate and elementary flows. Next, in Section 2.3, we outline the operationalization of the proposed framework, detailing how the mathematical framework can be integrated in common LCA and LCO modeling practices. Finally, Section 2.4 briefly introduces a Python-based implementation of the framework in the optimex package.

Throughout this work, we denote scalars by italic symbols, vectors by bold italic lowercase symbols, matrices by bold italic uppercase symbols, and sets by calligraphic uppercase symbols.

### 2.1 Foundation

In matrix-based LCA as formalized by Heijungs and Suh,^31^ environmental impacts ***h*** are calculated by characterizing a life cycle inventory ***g*** using a characterization matrix ***Q***

1 h=Qg

The life cycle inventory ***g*** is determined from the biosphere matrix ***B*** and the scaling vector ***s***

2 g=Bs

In turn, ***s*** is determined from the technosphere matrix ***A*** and the functional unit or demand vector ***f***

3 $$s=A^{-1}f$$

In LCO, the technosphere matrix ***A*** is not square, as multiple processes (i.e., columns) can produce the same products (i.e., rows).^16^ As a result, the system has degrees of freedom: different combinations of process scaling factors can satisfy the same product demand. These degrees of freedom can be exploited through optimization. Since only a square ***A*** is invertible, ***s*** cannot be determined directly from eq 3 in LCO. Instead, the scaling vector ***s*** is made the decision variable of an optimization problem, where ***s*** is determined such that the demand ***f*** is satisfied while minimizing or maximizing an objective function. Following Kätelhön et al.,^16^ a linear LCO problem with the objective to minimize environmental impact *h* ^c^, where *c* is a specific chosen impact category, can be formulated as

4a $$\min_{s}\;h^{c}=Q^{c}g=Q^{c}Bs$$

4b s.t.As=f

4c s≥0

### 2.2 Formulation

In the static LCO formulation described in eq 4a, neither temporal distribution nor temporal evolution of intermediate and elementary flows can be considered. In this section, we extend static to time-explicit LCO in four steps by (1) introducing explicit time indices and reflecting time lags through convolution, (2) enabling temporal evolution through vintage-dependent parameters, (3) differentiating installation- and operation-dependent flows, and (4) incorporating time-specific background systems. Finally, we compose the fully time-explicit LCO problem.

#### 2.2.1 Time-Explicit Matrices and Convolution

To make the static LCO formulation (eq 4a) time-explicit, we add time indices to its matrices and vectors. We introduce system time t∈T , representing absolute points in time (e.g., January 1, 2026). Several quantities from the static formulation naturally extend to system time: the demand vector ***f***_*t*_ specifies required product amounts at each point in time, the scaling vector ***s***_*t*_ represents process scaling decisions at each point in time, and the characterization matrix ***Q***_*t*_^c^ allows for time-specific characterization factors. Indexing the elements of the matrix formulation by absolute time in this way follows the dynamic LCA formulation proposed by Collinge et al.^19^

The technosphere and biosphere matrices ***A*** and ***B***, however, cannot simply be indexed by system time *t*. Their coefficients describe intermediate and elementary flows that are distributed across a process’s life cycle. This temporal profile is intrinsic to each process: regardless of when a process is installed, the relative timing of its flows remains the same. Since the installation time is itself a decision variable in LCO, expressing these profiles in absolute time would require specifying them anew for every point in time at which a process could be installed, and for every combination of installation times across processes. Instead, we introduce process time *δ* ∈ Δ, representing time relative to the installation of a process, departing from the purely absolute-time indexing of Collinge et al.^19^ The matrices ***A***_*δ*_ and ***B***_*δ*_ define what share of each flow occurs at what time delta relative to a process’s installation at *δ* = 0. For example, a process installed at *t* = 2025 can have its end-of-life treatment defined at *δ* = 10 years, translating to absolute time *t* = 2035. Figure 1 illustrates this mapping between process time and system time.

**Figure 1:**
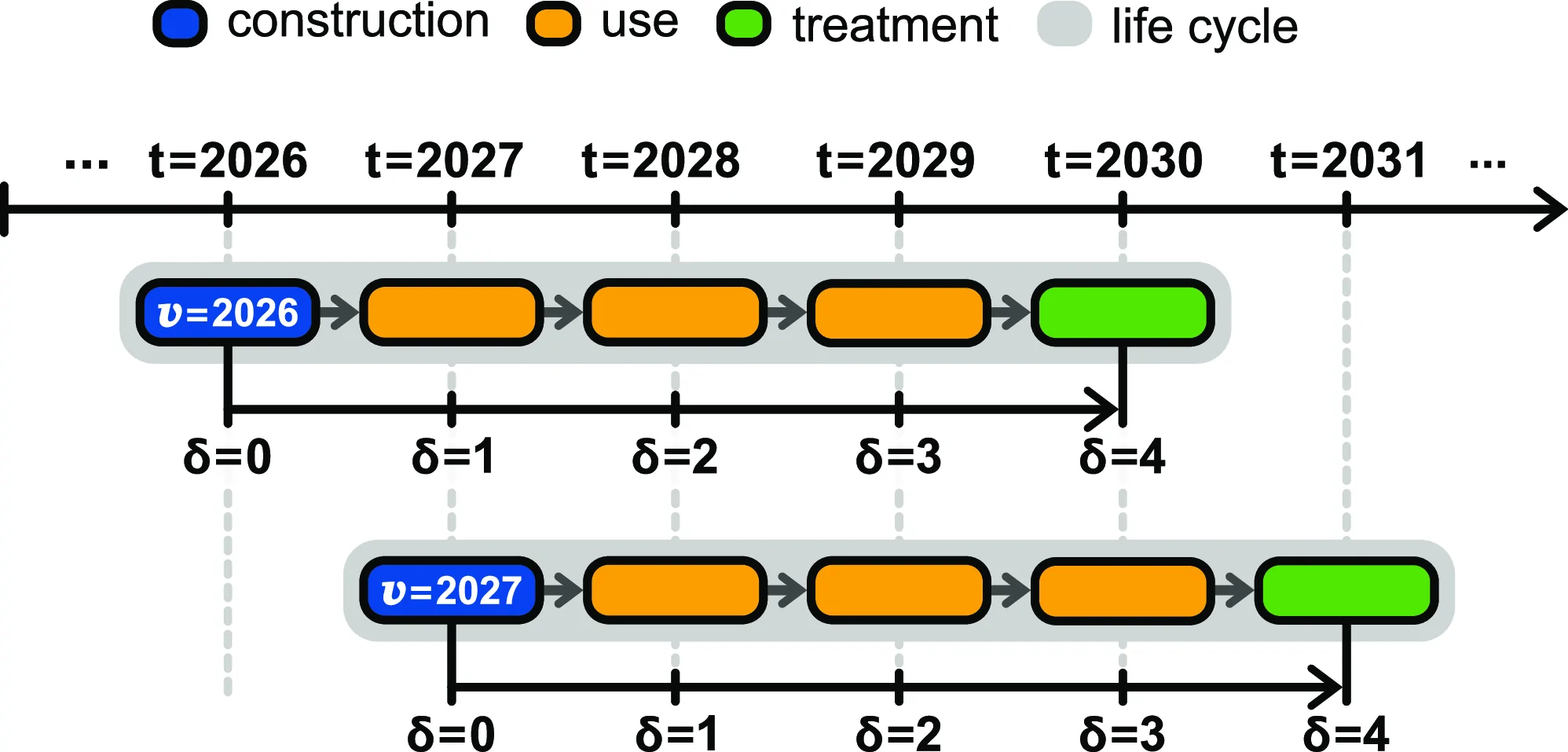
Relation between system time *t*, process time *δ*, and vintage *v*. Two vintages of the same process, installed at *v* = 2026 and *v* = 2027, each pass through construction at *δ* = 0, use at *δ* = 1 to 3, and treatment at *δ* = 4. Process time counts from the installation of the respective vintage, so that the same process time falls on different system times.

With ***A***_*δ*_ and ***B***_*δ*_ defined in process time and ***s***_*t*_ in system time, we need a mechanism to bridge the two temporal dimensions. Convolution achieves this by summing, for each system time *t*, over all processes whose life cycle overlaps with *t*

5a $${\left(A\times s\right)}_{t}=\sum_{\delta \in \Delta_{t}}A_{\delta }s_{t-\delta }$$

5b $${\left(B\times s\right)}_{t}=\sum_{\delta \in \Delta_{t}}B_{\delta }s_{t-\delta }$$

where $\Delta_{t}=\left\{\delta \in \Delta |t-\delta \in T\right\}$ is the subset of valid process time deltas at system time *t*. Convolving relative temporal profiles into absolute time is commonly used in time-resolved LCA models to place flows on a timeline.^18,20–22^ New here is that the convolution operates on matrices explicitly expanded by a relative time dimension, which is what allows the installation times to remain decision variables and be mapped to time-specific inventory data later on. The convolution yields the time-explicit inventory ***g***_*t*_, which captures all elementary flows occurring at each absolute point in time

6 $$g_{t}={\left(B\times s\right)}_{t}$$

Analogously, the demand satisfaction constraint ensures that intermediate flows balance at each point in time

7 $${\left(A\times s\right)}_{t}=f_{t}$$

The total environmental impact in a specific category *h* ^c^ is then obtained by summing the characterized inventory over all time steps

8 $$h^{c}=\sum_{t\in T}Q_{t}^{c}g_{t}$$

This formulation captures temporal distribution, i.e., the time lags between a process’s installation and its subsequent flows. However, ***A***_*δ*_ and ***B***_*δ*_ as defined here do not yet reflect temporal evolution: the coefficients are the same regardless of when a process is installed.

#### 2.2.2 Temporal Evolution through Vintage-Dependent Parameters

To capture temporal evolution, we note that the process-specific matrix coefficients of ***A*** and ***B*** are locked in at the moment of installation and do not change afterward. All units of a process installed at the same point in time therefore share the same coefficients over their entire operating life, forming a cohort commonly called a technology *vintage*. An electrolyzer built in 2030 and an electrolyzer built in 2040 are thus two vintages of the same process that operate side by side with different efficiencies. While tracking technologies by vintage is common in multi-period energy system models and IAMs^40–42^ and has also been integrated in time-resolved LCA,^43^ it has not seen integration in LCO. To achieve this integration, we introduce an additional vintage-time index v∈T , representing the installation time of each process unit. The individual coefficients of ***A*** and ***B*** become *a*_*p*,*i*,*δ*,*v*_ and *b*_*p*,*e*,*δ*,*v*_, respectively, where *p* denotes the process, *i* the intermediate flow, and *e* the elementary flow. Each coefficient thus depends on both process time *δ* and vintage *v*, such that a process installed at vintage *v* has its flows distributed according to *δ* and its flow magnitudes determined by *v*. The convolution from eq 5a generalizes to

9a $${\left(A\times s\right)}_{t}=\sum_{v\in V_{t}}A_{\delta =t-v,v}s_{v}$$

$${\left(B\times s\right)}_{t}=\sum_{v\in V_{t}}B_{\delta =t-v,v}s_{v}$$ 9b

Here, $V_{t}=\left\{v\in T\;|\;t-v\in \Delta \right\}$ is the set of vintages with flows occurring at system time *t*, determined by whether the process time *δ* = *t* – *v* falls within the defined time deltas Δ. For example, a process installed at *v* = 2025 evaluated at *t* = 2030 has *δ* = 5, meaning it is five years into its life cycle. The coefficient *a*_*p*,*i*,*δ*,*v*_ thus captures both the life cycle stage (via *δ* = *t* – *v*) and installation time (via *v*). The two vintages in Figure 1 illustrate this: both run through the same process times, but each starts counting at its own installation, so their life cycle stages fall on different system times.

To express technology evolution, we apply multiplicative scaling factors *ϕ*_*p*,*i*,*v*_ and *ϕ*_*p*,*e*,*v*_

10 $$a_{p,i,\delta ,v}=\phi_{p,i,v}\cdot a_{p,i,\delta }^{base},,b_{p,e,\delta ,v}=\phi_{p,e,v}\cdot b_{p,e,\delta }^{base}$$

where *a*_*p*,*i*,*δ*_^base^ and *b*_*p*,*e*,*δ*_^base^ are the respective base (vintage-independent) coefficients. A scaling factor *ϕ*_*p*,*i*,*v*_ = 0.7 indicates a 30% improvement relative to the base amount of the respective flow.

#### 2.2.3 Differentiating Installation- and Operation-Dependent Flows

So far, the scaling vector ***s*** scales all flows of a process jointly: installing one unit of capacity triggers both construction-related and operation-related flows in fixed proportion. In traditional LCA and LCO, this coupling is unavoidable because all flows are temporally aggregated. Because our formulation explicitly resolves time, we can distinguish between flows tied to the installation of a process (e.g., construction materials, end-of-life treatment) and flows tied to its operation (e.g., fuel consumption, operational emissions). This allows operational flows to scale independently of installed capacity: an electrolyzer’s electricity use can vary over time without affecting its construction footprint. To reflect this differentiation, we split the scaling vector into two components: capacity installation ***s***_*v*_ (indexed by vintage *v*) and operational level ***o***_*t*,*v*_ (indexed by system time *t* and vintage *v*). The entry *s*_*p*,*v*_ of ***s***_*v*_ states how many unit processes of process *p* are installed at vintage *v*, and thus how often the flows tied to the installation occur. The entry *o*_*p*,*t*,*v*_ of ***o***_*t*,*v*_ states how many of these installed unit processes are actually used at system time *t*. What changes compared to static LCA is at which system time *t* the flows of one unit process occur, as their rTDs spread them over process time *δ*. To clarify, consider an electrolyzer *p* as a unit process producing 1 kg of hydrogen, with a lifetime of ten time steps. In static LCA, installing one unit process of *p* at *t* = 2026 would result in 1 kg of hydrogen being produced at *t* = 2026. In contrast, in our time-explicit formulation, installing one unit process at *t* = 2026 (i.e., *s*_*p*,*v*=2026_ = 1) and operating it at full capacity throughout (i.e., *o*_*p*,*t*,*v*=2026_ = 1 for all *t* ∈ {2026, ..., 2035}) results in 0.1 kg of hydrogen being produced at each time step from *t* = 2026 to *t* = 2035, summing to 1 kg over the entire lifetime.

The underlying inventory data that determines the amounts of the time-distributed flows is split analogously. To differentiate which flows are scaled by ***s***_*v*_ and ***o***_*t*,*v*_, respectively, we first collect all operation-dependent intermediate and elementary flows in a set O . We then define separate matrices for operation (***A***_*v*_^op^, ***B***_*v*_^op^) and capacity installation (***A***_*δ*,*v*_^cap^, ***B***_*δ*,*v*_^cap^). The operation matrices ***A***_*v*_^op^ and ***B***_*v*_^op^ contain the coefficients per operated unit process and time step for flows in O , with vintage-specific values reflecting technology evolution (eq 10). The capacity matrices ***A***_*δ*,*v*_^cap^ and ***B***_*δ*,*v*_^cap^ contain all flows not in O , with the rest of the coefficients set to zero. Continuing the electrolyzer example, we could assume that the electrolyzer consumes 50 kWh of electricity per kg of hydrogen produced, requires 1 × 10^–7^ electrolyzers as construction input, and 1 × 10^–7^ used electrolyzers as end-of-life treatment input. Accordingly, the electrolyzer produces 0.1 kg of hydrogen per time step and consumes 5 kWh of electricity per time step, which are entries of ***A***_*v*=2026_^op^, applied at each *t* in which the electrolyzer is operated. The construction of 1 × 10^–7^ electrolyzers per unit process and their end-of-life treatment are entries of ***A***_*δ*,*v*=2026_^cap^, placed at the process times *δ* given by their rTDs (e.g., construction at *δ* = 0 and treatment at *δ* = 10), and triggered once by the respective entry of ***s***_*v*=2026_. Because operation can be scaled independently of installation, the entries of ***o***_*t*,*v*_ can reflect part-load behavior. For example, running the electrolyzer at half load in a given time step (*o*_*p*,*t*,*v*=2026_ = 0.5) yields 0.05 kg of hydrogen from 2.5 kWh of electricity at that *t*, while the construction and end-of-life flows stay as they are, since they scale with *s*_*p*,*v*=2026_ alone. The maximum operational level of a vintage is limited by its installed capacity

11 $$o_{t,v}\leq s_{v},\forall v\in V_{t}^{op}$$

The inventory ***g***_*t*_^flex^ now combines contributions from both capacity and operation matrices. Capacity-related flows (construction, end-of-life) can occur for any vintage *v* whose life cycle overlaps with *t*, captured by $V_{t}$ as before. Operational flows, however, can only occur while a process is still running. We restrict them to vintages within their operational lifetime by defining $V_{t}^{op}=\left\{v\in T|v\leq t\;\text{and}\;t-v\leq L_{p}\right\}$ , where *L*_p_ is the process-specific lifetime. The inventory becomes

12 $$g_{t}^{flex}=\sum_{v\in V_{t}}B_{\delta =t-v,v}^{cap}s_{v}+\sum_{v\in V_{t}^{op}}B_{v}^{op}o_{t,v}$$

The final demand ***f***_*t*_ is met by the sum of production across all operable vintages

13 $$\sum_{v\in V_{t}^{op}}A_{v}^{op}o_{t,v}=f_{t}$$

#### 2.2.4 Incorporating Background Systems

The formulation developed so far is self-contained and can be used for bottom-up system modeling. However, real-world supply chains are complex, making fully bottom-up modeling impractical. In LCA and LCO practice, the system is therefore commonly divided into a foreground system and a background system.^44^ The foreground comprises the processes that are directly modeled and subject to optimization, while the background represents the broader economy and upstream supply chains that lie outside the decision maker’s scope of influence. Because background systems cannot directly be influenced, their supply chains are fixed and contain no decision variables in the LCO context, and can therefore be sourced from life cycle inventory databases.

To incorporate background systems, we partition the technosphere into foreground ***A*** ^fg^ and background ***A*** ^bg^, and correspondingly the biosphere into ***B*** ^fg^ and ***B*** ^bg^. The formulation from the preceding sections applies to the foreground, with ***g***_*t*_^fg,flex^ following directly from eq 12. For the background, we need to determine the inventory induced by the foreground’s demand for background processes.

##### 2.2.4.1 Background Inventory

The foreground’s intermediate flows to the background define the demand for background processes at time *t*

14 $$f_{t}^{bg}=\sum_{v\in V_{t}}A_{\delta =t-v,v}^{cap}s_{v}+\sum_{v\in V_{t}^{op}}A_{v}^{op}o_{t,v}$$

***f***_*t*_^bg^ acts as a model-internal demand for a separate background calculation that does not require an additional input. Instead, ***f***_*t*_^bg^ follows from the capacity and operation variables and thus, ultimately, from the demand ***f***_*t*_ that the practitioner specifies. Separating it from the foreground only serves to keep the background sourceable from life cycle inventory databases rather than requiring it to be modeled explicitly.

The elementary flows per unit product from a background process are captured by **Λ**, the intensity matrix^31^

15 $$\Lambda =B^{bg}{\left(A^{bg}\right)}^{-1}$$

Notably, ***A*** ^bg^ is a square matrix (i.e., each product is produced by exactly one process in the background), thus it is invertible and contains no decision variables for the optimization.

In the simplest case, **Λ** is static and the background inventory at time *t* is

16 $$g_{t}^{bg}=\Lambda f_{t}^{bg}$$

##### 2.2.4.2 Temporal Evolution through Time-Specific Background Systems

A static **Λ** does not reflect that background supply chains evolve over time. To capture this, we allow demands to be fulfilled from different time-specific background systems versions b∈B , each representing the state of the economy at a particular point in time. A mapping matrix ***M*** assigns system times *t* to background system version *b*: in the simplest case, ***M*** is an identity matrix. When background system versions are available at coarser resolution (e.g., 5 year steps) while system time *t* is finer resolution, ***M*** can interpolate between available versions. The background inventory from eq 16 then generalizes to

17 $$g_{t}^{bg}=\left(\sum_{b\in B}\Lambda_{b}M_{b,t}\right)f_{t}^{bg}$$

##### 2.2.4.3 Temporal Distribution through Graph Traversal

Equation 17 assumes that all upstream background processes triggered by a foreground demand at time *t* also occur at *t*. In reality, background supply chains also encompass temporal distribution: an intermediate flow in the background may occur at a time-shifted point *t* + *δ*′, where a different background system version may apply, cascading into different elementary flow amounts upstream. To jointly capture both temporal evolution and distribution in the background, we define a temporally resolved intensity matrix ${\mathrm{\Lambda ̂}}_{t}$

18 $$g_{t}^{bg,flex}={\mathrm{\Lambda ̂}}_{t}f_{t}^{bg}$$

To construct ${\mathrm{\Lambda ̂}}_{t}$ , we traverse the background system supply chains, treating them as a weighted directed graph, where time deltas *δ* can be defined for each edge, i.e., each intermediate or elementary flow.^22,45^ For each background intermediate flow demanded at system time *t*, the traversal checks if a time delta *δ*′ is defined for the specific flow, computes the absolute system time *t*_*n*_ = *t* + *δ*′, and uses ***M*** to select the appropriate time-specific background system version to identify the next node to traverse to. When a time lag *δ*′ is defined on a background intermediate flow, the upstream node is visited at *t*_*n*_ + *δ*′, shifting which time-specific background system version applies. When no temporal distributions are defined, all upstream nodes are evaluated at the same *t* and the traversal reduces to eq 17.

##### 2.2.4.4 Combined Inventory and Impact

With both foreground and background inventories determined, the environmental impact at time *t* is

19 $$h_{t}^{c}=Q_{t}^{c}\left(g_{t}^{fg,flex}+g_{t}^{bg,flex}\right)$$

#### 2.2.5 Complete Optimization Problem Formulation

Combining all elements, the complete time-explicit LCO problem is

20a $$\min_{s_{v},o_{v,t}}\;\sum_{t\in T}Q_{t}^{c}\left(g_{t}^{fg,flex}+g_{t}^{bg,flex}\right)$$

20b $$s.t.\;g_{t}^{fg,flex}=\sum_{v\in V_{t}}B_{v,\delta =t-v}^{cap}s_{v}+\sum_{v\in V_{t}^{op}}B_{v}^{op}o_{v,t}\;\;\;\forall t\in T$$

20c $$g_{t}^{bg,flex}={\mathrm{\Lambda ̂}}_{t}f_{t}^{bg},\forall t\in T$$

20d $$f_{t}^{bg}=\sum_{v\in V_{t}}A_{v,\delta =t-v}^{cap}s_{v}+\sum_{v\in V_{t}^{op}}A_{v}^{op}o_{v,t}\;\;\;\forall t\in T$$

20e $$o_{v,t}\leq s_{v},\forall t\in T,v\in V_{t}^{op}$$

20f $$\sum_{v\in V_{t}^{op}}A_{v}^{op}o_{v,t}=f_{t},\forall t\in T$$

20g $$s_{v}\geq 0\;\;\;\forall v\in T,\;\;\;o_{v,t}\geq 0\;\;\;\forall t\in T,v\in V_{t}^{op}$$

### 2.3 Operationalization

We now describe how to operationalize the proposed mathematical framework for time-explicit LCO, detailing the steps required to implement the framework in practice (see Figure 2). First, we explain how foreground systems are modeled, including the temporal information, the differentiation of installation- and operation-dependent flows, and vintage-specific evolution (Section 2.3.1). Next, we detail how time-specific values are inserted into time-explicit matrices (Section 2.3.2). Then, we outline how the final optimization problem is composed (Section 2.3.3). Finally, we describe how the problem is solved and what the solution returns (Section 2.3.4).

**Figure 2:**
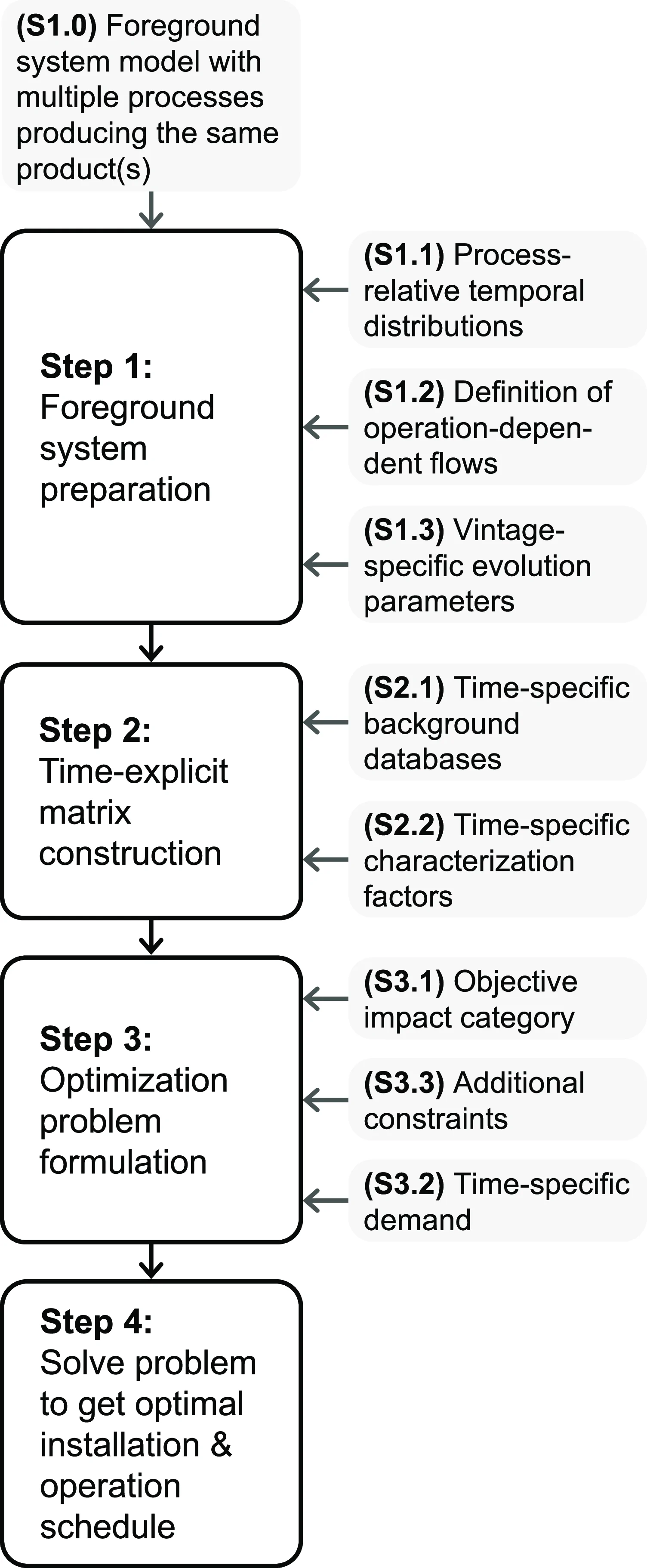
Time-explicit LCO workflow.

#### 2.3.1 Step 1: Foreground System Preparation

Step 1 of a time-explicit LCO is the foreground system preparation. As in conventional LCO, this begins with modeling the product system by defining products, processes, and their intermediate and elementary flows (S1.0 in Figure 2). This product system can include multiple processes producing the same product(s), e.g., reflecting different technologies or different process locations. Building on this product system model, the preparation then involves three additional tasks. First, we embed temporal information in the product system model, reflecting the temporal distributions of intermediate and elementary flows (S1.1). To achieve this, process-relative temporal distributions (rTDs) are used, following the approach introduced by Beloin-Saint-Pierre et al.^20^ and implemented by Cardellini et al.^22^ These rTDs define what share of an intermediate or elementary flow occurs at what time delta, with respect to the consuming or emitting process. The underlying temporal resolution of rTDs is flexible, and can range from hourly to yearly values. Second, we define operation-dependent flows so they can be scaled separately from capacity installation (S1.2). Third, to reflect the temporal evolution of foreground processes through vintage-dependent parameters (cf. eq 10), technology evolution can be specified as a mapping from absolute system times *t* to time-specific scaling factors *ϕ* for each intermediate and elementary flow (S1.3).

#### 2.3.2 Step 2: Time-Explicit Matrix Construction

In step 2, the temporalized foreground system from the previous step is combined with two additional inputs, time-specific background databases (S2.1) and time-specific characterization factors (S2.2), to construct the full set of time-explicit matrices (Figure 2).

For the foreground, the rTDs, operation-dependent flow definitions, and vintage-specific evolution parameters are translated into the time-explicit technosphere and biosphere matrices. The rTDs determine the distribution of each flow across process time steps *δ*. The operation-dependent flow classification splits these into capacity matrices (***A***_*δ*,*v*_^cap^, ***B***_*δ*,*v*_^cap^) and operation matrices (***A***_*v*_^op^, ***B***_*v*_^op^). The vintage-specific evolution parameters are then applied to the corresponding coefficients.

For the background, time-specific background databases (S2.1) representing the state of background production systems at specific absolute points in time are used to construct the intensity matrices **Λ**_*b*_ (eq 15). These databases can be created based on own assumptions or on external scenarios, e.g., using tools like premise.^25^ The temporal resolution of the background databases is not required to match the temporal resolution of the rTDs, as the mapping matrix ***M*** bridges the two resolutions.

Finally, time-specific characterization factors (S2.2) populate the characterization matrices ***Q***_*t*_. The framework is agnostic to the source of these factors and applicable across impact categories where timing matters, including climate change, water use,^46^ toxicity,^47^ or noise.^48^ For climate change, for example, dynamic LCIA methods can provide dynamic metrics such as radiative forcing (RF), absolute global warming potential (AGWP, i.e., the cumulative RF over time), or absolute global temperature change potential (AGTP), or prospective-dynamic metrics such as prospective AGWP and AGTP as recently proposed by Barbosa Watanabe and Cherubini.^26^ Alternatively, static characterization factors can be used when temporal differentiation is not required for a given impact category.

#### 2.3.3 Step 3: Optimization Problem Formulation

To formulate the optimization problem in step 3, we first choose an objective impact category (S3.1), which can be any environmental impact category and metric. Next, we define a time-specific demand (S3.2), specifying product amounts and the points in time at which they are required. This demand can be expressed as an rTD with respect to a user-defined (absolute) reference time. Together with the time-explicit LCA matrices introduced in step 2, these inputs parameterize the optimization model in eq 20a. Optionally, additional constraints can be formulated (S3.3), such as existing (brownfield) process capacities and stock- or flow-related limits on process installations, environmental impact categories, or individual intermediate or elementary flows. As all these quantities are indexed by system time, a limit can be placed either on the flow amount at each time step, yielding a flow-related limit, or on its cumulative sum over the horizon, yielding a stock-related limit.

#### 2.3.4 Step 4: Solving the Optimization Problem

The problem formulated in step 3 (eq 20a) is a linear program, as both the objective and all constraints are linear in the decision variables ***s***_*v*_ and ***o***_*t*,*v*_. Step 4 therefore amounts to solving a linear system of equations and inequalities, which can be done to global optimality with any linear programming solver.^49^

The solution returns the optimal entries of the capacity installation ***s***_*v*_ and the operational level ***o***_*t*,*v*_. Together, they constitute the transition pathway, i.e., how many unit processes of each process are installed at each vintage *v*, and how many of these are operated at each system time *t*. Beyond the objective value, inserting the optimal ***s***_*v*_ and ***o***_*t*,*v*_ back into eqs 12, 14, and 18 returns the foreground and background inventories ***g***_*t*_^fg,flex^ and ***g***_*t*_^bg,flex^ at every point in time, and with them the time-specific impacts *h*_*t*_^c^ (eq 19). As all these quantities are indexed by vintage and system time, they can be resolved by process, vintage, and impact category, so that a pathway can be retrospectively inspected for when emissions and resource extractions occur, which vintages cause them, and how closely stock- and flow-related limits are approached over the transition period.

### 2.4 Implementation

The proposed time-explicit LCO framework is implemented in the open-source Python package optimex.^50^ For LCA-related capabilities, optimex builds on the open-source LCA framework Brightway.^38^ Temporal information is encoded in the product system model in the same way as in bw_timex,^51^ the Brightway package for time-explicit LCA: rTDs are stored directly with the intermediate and elementary flows of the underlying database. Temporalized product system models are therefore fully compatible between the two packages, so that the same model can be assessed with bw_timex and optimized with optimex. For dynamic LCIA, optimex directly sources methods from the Brightway library dynamic_characterization.^52^ For optimization, optimex uses the open-source optimization framework pyomo.^39^ Additional information and instructions are available in the comprehensive online documentation of optimex.^53^ Together, these components allow practitioners to set up time-explicit LCO problems using familiar LCA and optimization workflows.

## 3 Application

To demonstrate the capabilities of time-explicit LCO, we apply the proposed framework to an integrated industrial system co-producing methanol and pig iron through a combination of conventional and novel technologies. This case study illustrates methodological capabilities rather than comprehensively assessing methanol and pig iron production systems. Section 3.1 describes the production system and optimization setup following the previously described workflow (see Figure 2). Section 3.2 presents the case study results. The full case study code is available as an annotated Jupyter Notebook in the optimex GitHub repository.^50^

### 3.1 Case Study Setup

As the foreground system that is subject to optimization, we consider a production system for methanol and pig iron (Figure 3).

The production system must meet a constant annual demand of 1 Mt methanol and 1 Mt pig iron from 2025 to 2050. We model this as a brownfield optimization problem, accounting for existing fossil-based production infrastructure. We assume that 0.5 Mt of yearly capacity was installed in both 2005 and 2015 for both methanol (via natural gas reforming) and pig iron (via standard blast furnace), totaling 1 Mt of legacy capacity at the start of the transition period.

From the beginning of the transition period in 2025, the system can draw on additional process and supply chain choices to satisfy the demands. For methanol synthesis, the model can choose between conventional natural gas (NG) reforming (lifetime *L* = 25 years) and CO_2_ hydrogenation (*L* = 15 years). The latter offers flexible CO_2_ sourcing, either from point-source capture via a blast furnace with carbon capture (BF-CC) or from atmospheric removal via direct air carbon capture (DAC). Similarly, pig iron production is diversified through standard blast furnaces, BF-CC, and hydrogen-based direct reduction of iron (H_2_-DRI). These production routes are highly intertwined: the BF-CC process co-produces pig iron and the CO_2_ feedstock for methanol synthesis, while proton exchange membrane (PEM) electrolysis (*L* = 8 years) provides the hydrogen required for both CO_2_ hydrogenation and the H_2_-DRI route. Each process is associated with specific installation- and operation-dependent intermediate and elementary flows, distributed across distinct time-relative phases: construction (*δ* ≤ 0), operation (*δ* ∈ [0, *L*]), and end-of-life treatment (*δ* > *L*). We derive process-specific inventories from the ecoinvent 3.12 database.^54^

With the foreground system defined, we specify the optimization objective and LCIA methods. We minimize climate impacts, assessed through the dynamic AGWP with a fixed end year of 2125 for all emissions. The dynamic AGWP corresponds to the cumulative RF over time, i.e., the total energy commitment to the atmosphere caused by the inventory. For further analysis of climate impacts, we additionally assess the underlying instantaneous RF. This instantaneous RF represents the actual warming rate at a specific point in time, including the effect of previous emissions that have partially decayed until then. As an additional impact category, we consider the water use impact, which we assess through static characterization factors from Environmental Footprint 3.1.^55^

To demonstrate the framework’s versatility, we define three modeling scenarios. The “Baseline” scenario has no temporal evolution, i.e., foreground parameters and background supply chains are fixed at 2025 states. The “Evolution” scenario assumes continuous improvement in both the foreground and the background system. In the foreground, efficiency gains are modeled through vintage-dependent scaling factors, including decreasing electricity consumption for PEM electrolysis, decreasing electricity and heat consumption for DAC, and decreasing electricity consumption and reduced direct fugitive CO_2_ emissions for the CO_2_ hydrogenation process. For the background system, we assume extensive decarbonization. We utilize a set of prospective ecoinvent 3.12 databases^54^ modified according to the REMIND-EU IAM^56^ in the SSP2-NDC scenario. This scenario reflects “middle of the road” socioeconomic developments with climate targets following Nationally Determined Contributions (NDCs), leading to below 2.5 °C warming by 2100. The prospective databases cover the period from 2020 to 2100 and are created via premise (version 2.3.4).^25^ Finally, the “Constrained Evolution” scenario introduces resource bottlenecks on top of the Evolution scenario, including a flow-related annual limit on water use impact (3 × 10^5^ m^3^-eq) and a stock-related cumulative limit on iridium extraction (0.125 kg) as a proxy for critical mineral scarcity.

Beyond these three scenarios, we assess the value of anticipating temporal evolution at the planning stage. To this end, we compare two designs under the same evolving conditions of the Evolution scenario. The first is the design optimized directly for the Evolution scenario, representing a planner that anticipates future change. The second is the design optimized for the Baseline scenario, representing a planner that assumes no evolution and locks in the design. In the latter case, the Baseline design is held fixed and its impacts are evaluated as the underlying system evolves according to the Evolution scenario, i.e., we conduct a time-explicit LCA for the Baseline design in the Evolution scenario. Comparing both designs under identical evolving conditions isolates the penalty of planning without anticipating evolution.

**Figure 3:**
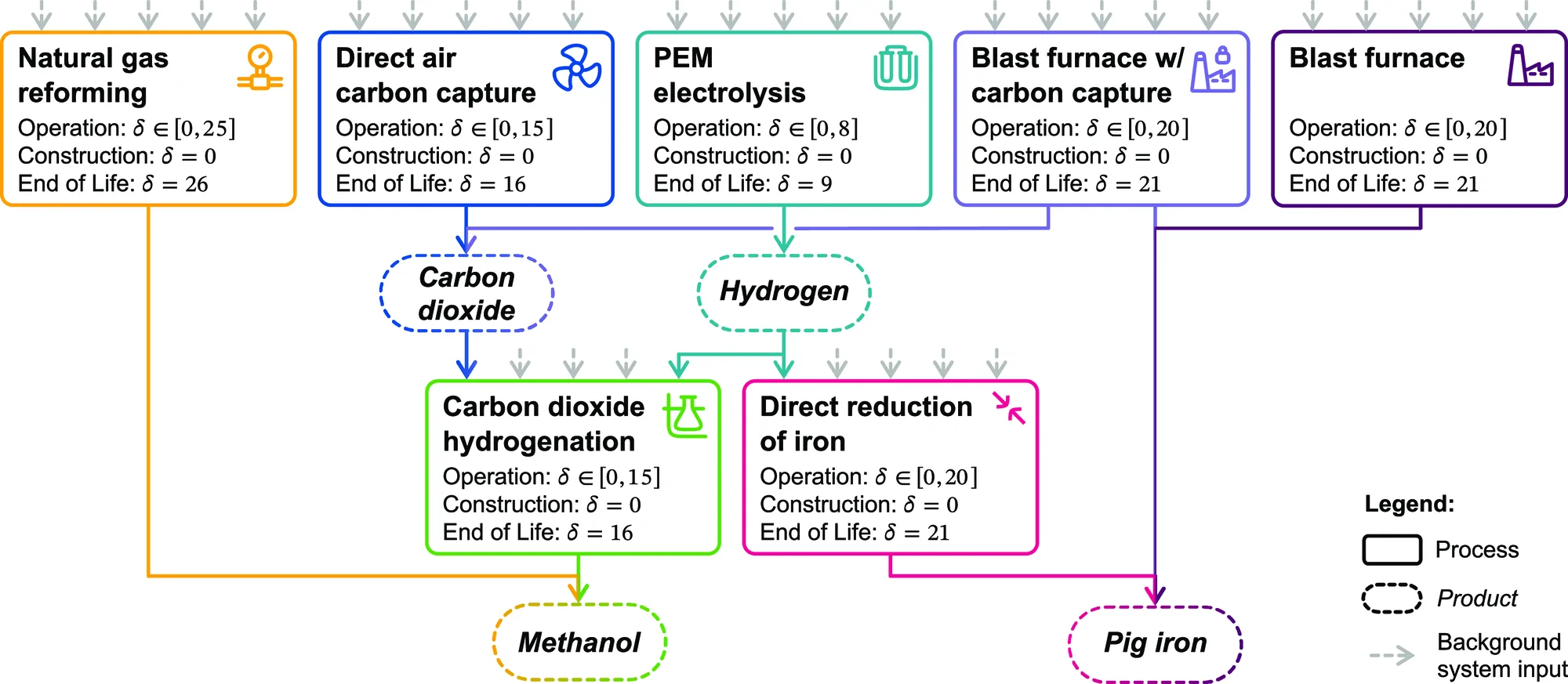
Production system for methanol and pig iron.

We solve the resulting optimization problems to global optimality with the Gurobi solver.^57^ All further modeling details and assumptions are available in an annotated Jupyter Notebook in the optimex GitHub repository.^50^

### 3.2 Results

The time-explicit LCO results for the integrated methanol and pig iron production system across scenarios are summarized in Figure 4.

In the Baseline scenario (Figure 4a-1 to g-1), the model identifies no incentive for technological change, maintaining fossil-based natural gas reforming and blast furnaces through 2050, which yields a total AGWI of 4.63 × 10^–6^ W m^–2^ yr. The installed capacity (dashed lines) exactly matches the demand, representing 100% utilization of installed capacity. Once installed capacities reach the end of their lifetime, new capacities are immediately installed to replace them. Figure 4e-1 shows the resulting climate impact through the annual Absolute Global Warming Impact (AGWI). The AGWI bars represent the warming impact of the emissions occurring in each year, integrated over the remaining time until 2125. For the Baseline scenario, the annual AGWI declines over time. This decline is a consequence of the fixed 2125-time horizon: later emissions contribute to warming over a shorter remaining duration, so their impact is effectively reduced. In contrast, the instantaneous RF curves (f-1) depict the actual warming rate caused by the inventory. While these curves rise, their slope flattens over time. This flattening is driven by the atmospheric decay of greenhouse gases: as the inventory accumulates, the radiative forcing lost to the decay of past emissions increasingly counteracts the forcing added by new emissions, causing the net growth rate to slow. For water use, the impact remains constant throughout the period (g-1) because we use static characterization factors.

In the Evolution scenario (Figure 4a-2 to g-2), the model identifies a phased transition toward electrification starting in 2033, cutting the total AGWI to 1.82 × 10^–6^ W m^–2^ yr, which is 61% below the Baseline scenario. As background electricity impacts decrease, the system switches to CO_2_ hydrogenation for methanol and H_2_-DRI for pig iron. Overcapacities emerge (dashed lines exceeding solid bars): the model finds that switching earlier to electrification routes, and the resulting savings in operational emissions, outweighs the impacts of idled fossil-based brownfield capacities. Beyond these capacity decisions, the scenario reveals how technology improvement shapes climate trajectories (Figure 4e-2,f-2). For instance, for electrolysis, even when production levels remain constant after 2033 (d-2), the yearly AGWI bars decline due to the decarbonization of background inputs and efficiency improvements. Compared to the Baseline scenario (e-1), where the decline stems solely from the decreasing integration horizon, the Evolution scenario shows a more pronounced decrease. Accordingly, the slope of the corresponding instantaneous RF curves (f-2) also decreases as cleaner production vintages are preferentially operated later, idling previously built and still operable installations.

**Figure 4:**
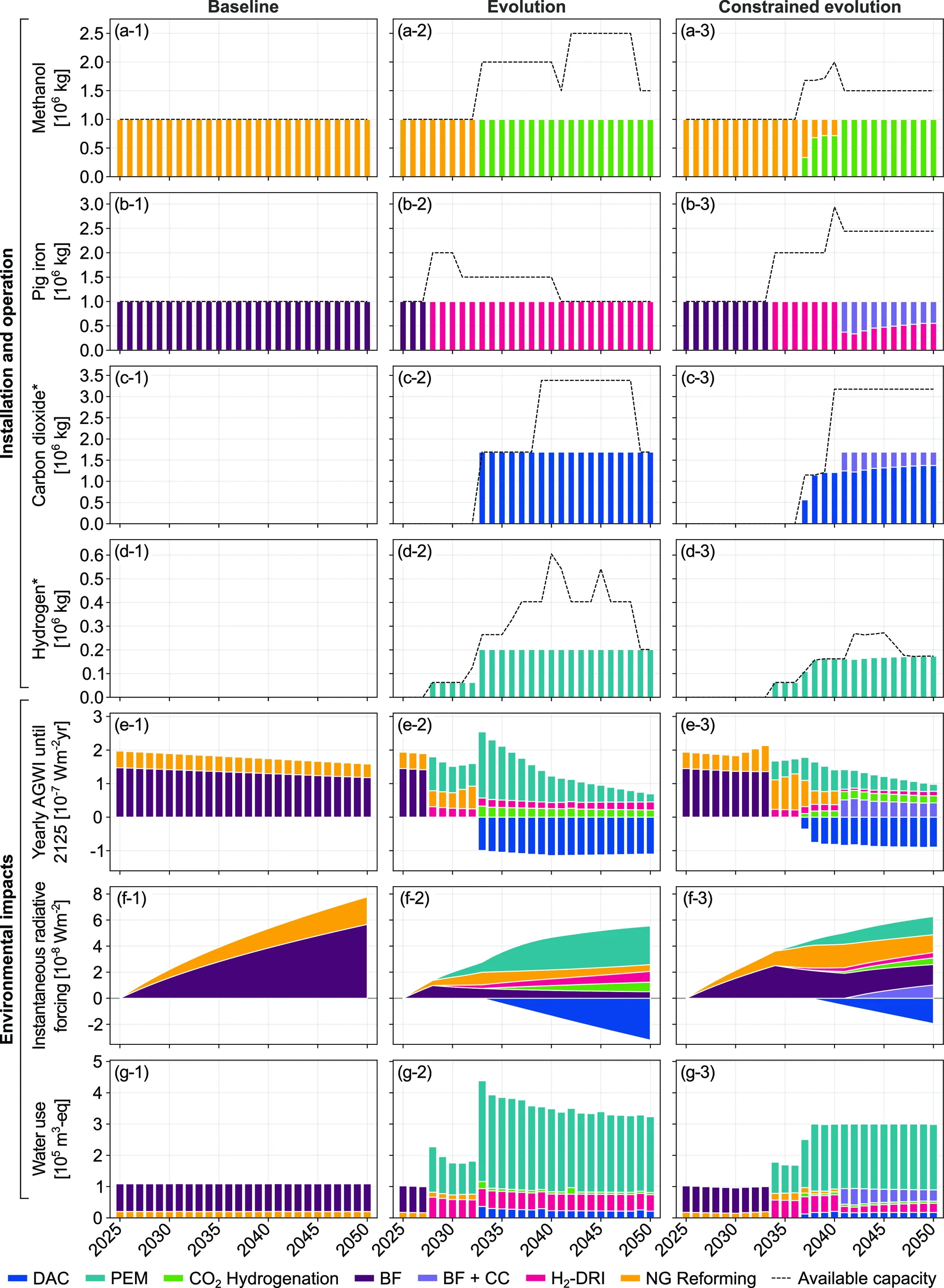
Installation, operation and environmental impacts of the optimized methanol and pig iron production system over the transition pathway. Asterisks indicate intermediate products.

Notably, the DAC route contributes a negative AGWI in several years (e-2). Because CO_2_ hydrogenation draws its CO_2_ from DAC, the captured atmospheric CO_2_ enters the inventory as a negative elementary flow, i.e., a cooling (negative RF) contribution. As the background decarbonizes, the upstream impact of powering DAC falls while its CO_2_ uptake stays fixed, so the DAC’s contribution grows increasingly negative until 2040, after which the shrinking remaining horizon until 2125 reduces the impact again. From 2042 onward, the DAC cooling outweighs the residual positive contributions, and the system-wide yearly AGWI itself turns negative (e-2). Consequently, the flows of those years commit the atmosphere to net cooling over the remaining horizon. The instantaneous RF, in contrast, stays positive throughout (f-2). While the yearly AGWI integrates only *that year’s own flows*, turning negative once these flows cause net-negative forcing, the instantaneous RF reflects the forcing actually present at that moment, i.e., the accumulated stock from *all preceding years*. Since the early transition years cause net-positive emissions and greenhouse gases decay slowly, this stock stays large and positive even after annual flows turn net-removing.

Finally, the Constrained Evolution scenario (Figure 4a-3 to g-3) demonstrates the framework’s ability to navigate resource bottlenecks. The previous evolution scenario revealed that hydrogen-based routes carry substantial water impacts (g-2). By imposing annual water availability caps and a cumulative limit on iridium, which mostly affect the PEM electrolyzer, the model is forced to diversify its technological portfolio. The system shifts back toward a mix of technologies in later years, utilizing BF-CC for iron production to stay within resource limits, but raising the total AGWI to 2.93 × 10^–6^ W m^–2^ yr, 61% above the unconstrained Evolution optimum, yet still 37% below the Baseline scenario. Both the water use and the iridium limits act on the same time-explicit inventory, in which the elementary flows of all vintages and their supply chains are collected at the time step in which they occur, but they constrain it differently. The water use cap applies to the inventory at each individual time step and thus binds whenever the annual water use would exceed it (g-3). The iridium limit applies to the sum of the inventory over the entire horizon and is therefore not tied to any single point in time. Since the inventory covers all processes and all supply chain levels, this bounds the total iridium extraction of the system rather than the demand of any single process, reflecting the system-wide nature of critical mineral scarcity. Within this limit, the model is free to allocate the available iridium to the processes and points in time at which it yields the largest benefit.

Together, the three scenarios describe the design that is optimal under each set of assumptions, but they do not reveal how much is lost by planning without anticipating evolution at all. Figure 5 isolates this penalty by comparing the optimized design in the evolution scenario with the baseline design that was optimized without anticipating evolution, but the resulting impacts are then evaluated considering evolution. The non-anticipating Baseline design causes 142% higher AGWI (4.41 × 10^–6^ versus 1.82 × 10^–6^ W m^–2^ yr) than the design optimized directly for the Evolution scenario. The two trajectories also show where this gap originates. In the early years, both designs install and operate the same fossil-based capacities (cf. Figure 4), so their impacts coincide. They diverge once the design optimized for the Evolution scenario begins its transition toward electrified routes in 2033, while the Baseline design remains locked into its fossil-based configuration. For reference, the Baseline design evaluated entirely without evolution, i.e., in the Baseline scenario, lies even higher at 4.63 × 10^–6^ W m^–2^ yr, since under the Evolution scenario even the fossil-based supply chains decarbonize slightly and thereby marginally lower the impacts of the otherwise unchanged design. Anticipating evolution during planning therefore reveals lower-impact pathways, as the optimization favors designs that remain advantageous as the system evolves rather than locking in choices that appear optimal only for a static, present-day system model.

**Figure 5:**
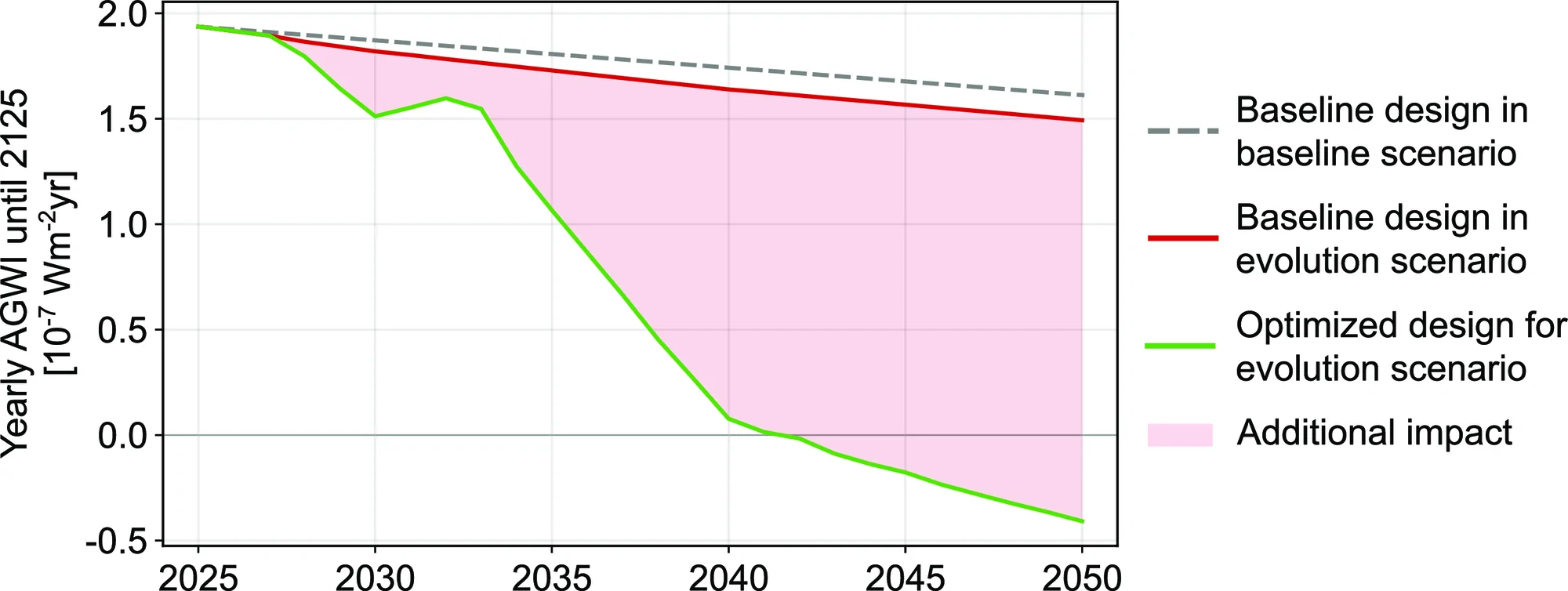
Effect of anticipating temporal evolution during optimization. Yearly AGWI of the Baseline design and of the design optimized for the Evolution scenario. The Baseline design is optimized without anticipating evolution, then held fixed and evaluated under the evolving conditions. The Baseline design in the Baseline scenario is shown for reference.

Overall, these results illustrate that time-explicit LCO identifies transition pathways that navigate complex temporal interdependencies hidden in static assessments. By resolving these interdependencies, the framework reveals trade-offs such as strategic overcapacities with preferential operation of cleaner vintages, and navigates transient resource bottlenecks through technology diversification. Anticipating evolution at the planning stage moreover yields designs that stay advantageous as the system develops. These capabilities remain inaccessible to models that collapse all flows to a single point in time.

## 4 Discussion

Designing sustainable transition pathways that respect environmental constraints requires optimization models capable of capturing when exchanges with the environment occur, not just their aggregated magnitudes. The time-explicit LCO framework presented in this work addresses this requirement by integrating the temporal distribution of life cycle stages and evolution of production systems into optimization models. Whereas existing LCO approaches either neglect time entirely or only account for prospective changes in background systems, our framework tracks the full temporal profile of both intermediate and elementary flows across life cycles and supply chains, while simultaneously reflecting technological progress and economy-wide decarbonization.

### 4.1 Relevance of Time-Explicit Life Cycle Optimization

The case study (Section 3.2) demonstrates how the interplay between installation timing, operational emissions, and technological evolution leads to transition pathways that differ substantially from static approaches. Capabilities inaccessible to static approaches emerge: the framework identifies hidden trade-offs, such as strategic overcapacities where early investment in cleaner technologies outweighs embodied infrastructure impacts and newer vintages are preferentially operated, and navigates transient resource bottlenecks by diversifying technology portfolios. The comparison of the Baseline and Evolution designs under identical evolving conditions quantifies the benefit of this foresight, as the design that anticipates evolution reaches less than half the climate impact of the design that does not. Anticipating evolution is therefore not only a matter of assessment accuracy but changes which pathway is selected, which matters wherever long-lived capacities are planned against rapidly evolving supply chains.

These capabilities depend not only on temporal resolution in the inventory but also on the choice of impact assessment method. The framework supports both static and dynamic LCIA, with the latter necessary for capturing time-sensitive impacts. For climate change, dynamic metrics such as RF are essential when considering the different atmospheric lifetimes of greenhouse gases and their time-dependent energy absorptions. Beyond climate change, the framework extends to other impact categories where timing matters, such as water use,^46^ toxicity,^47^ or noise.^48^

Time-explicit LCO is broadly relevant across sectors where temporal dynamics are decisive, for example: (1) systems depending on evolving upstream processes, where inputs such as electricity, steel, or hydrogen undergo rapid decarbonization; (2) early-stage technologies with steep learning curves, where vintage-dependent performance gains are significant; (3) circular economy planning with long material residence times, where decades-long lags between installation and end-of-life create temporal mismatches between primary demand and secondary supply; (4) time-resolved carbon accounting, such as biogenic feedstocks with varying rotation periods, temporary carbon storage in CO_2_-based products, or carbon dioxide removal technologies with varying temporal profiles for CO_2_ removals; (5) the built environment, where long service lives create substantial temporal mismatches between construction, operation, and end-of-life impacts; and (6) multi-regional supply chains with divergent evolution, where sourcing decisions must account for heterogeneous decarbonization trajectories across production regions alongside region-specific total availabilities. Across these applications, the framework’s temporal resolution is fully flexible: while the case study demonstrates yearly resolution, seasonal resolution can capture water availability constraints, and hourly resolution can reflect the diurnal availability of renewable electricity.

To facilitate the application of time-explicit LCO, we provide an open-source implementation in the Python package optimex,^50^ building on the Brightway LCA framework^38^ and the pyomo optimization framework.^39^ Users can leverage familiar LCA modeling approaches to define production systems, and then extend them with rTDs, vintage-dependent evolution factors, and the classification of installation and operation-dependent flows, enabling independent scaling of infrastructure required for vintage-specific process operation. By natively integrating time-specific databases from premise^25^ and dynamic characterization functions from dynamic_characterization,^52^ optimex automatically constructs the optimization model from these inputs, allowing practitioners to focus on process and system modeling.

### 4.2 Practical Considerations and Challenges

Applying time-explicit LCO raises practical questions that static LCO does not face, concerning the representation of time in the model, the underlying data, the computational cost, and the generalizability of the resulting pathways.

To represent time in the proposed time-explicit LCO framework, we split the time dimension across three indices, each carrying a different property of a flow. Process time *δ* places a flow relative to the installation of its process, the vintage *v* fixes its magnitude at the performance level of that technology generation, and system time *t* places it in absolute time, with a convolution linking the three (eq 9a). Keeping them apart is a deliberate choice that limits the modeling effort. Relative timing and vintage-dependent performance are properties of a process, so the rTDs and scaling factors *ϕ* are defined once per process, independently of when and how often that process is later installed. The number of inputs therefore scales with the number of processes, and not with the number of processes times the number of time steps.

This representation of time diverges from previous approaches. In the time-explicit LCA framework by Müller et al.,^18^ an absolute timeline of intermediate flows is constructed by cascading rTDs throughout the product system. Based on this timeline, the traditional LCA matrices are expanded with additional row–column pairs, so that each flow at a specific absolute time becomes a separate matrix element. The supply chain LCO model of Hülagü et al.^33^ populates a complete time-specific matrix for every period of the planning horizon. These two approaches presuppose that the absolute time of every flow is known beforehand, which holds in time-explicit LCA, where the timeline of intermediate flows for a given time-specific demand fixes the timing of all flows. In time-explicit LCO, however, installation times are decision variables. Reproducing the matrix expansion would therefore require specifying the time-shifted flows of a process once for every point in time at which it could be installed, and doing so for every combination of installation times across the processes of the pathway. Since temporal shifts also occur at multiple levels of the upstream supply chains, each such specification cascades further, which makes the pre-enumeration impractical precisely because these combinations are part of the solution space. This difficulty does not arise in the model of Hülagü et al.,^33^ as their single installation decision at the start of the horizon fixes the absolute timing of all flows beforehand, so that manually specifying them in absolute time remains feasible and time-indexed matrices remain sufficient. The convolution avoids the pre-enumeration by deferring the placement of flows in absolute time to the optimization itself, and is therefore what makes a transition with multiple installation times tractable in the first place.

Time-explicit LCO inherits the data requirements of time-explicit LCA and adds several of its own. Shared with time-explicit LCA is the need for temporal distributions, time-specific inventory data, and time-explicit impact assessment methods. Practitioners can usually estimate rTDs for their foreground system. Temporalizing the background system is more demanding and requires systematic procedures, such as the supply-delay framework proposed by Pigné et al.,^58^ which come at a high computational cost. Prospective background data generation tools such as premise^25^ have made time-specific LCI data for climate change mitigation scenarios readily available, while time-specific data for elementary flows relevant to other impact categories or at higher temporal resolution remain scarce. Historical LCA^59^ improves the coverage of past time steps, and real-time inventory data are gaining traction in the electricity sector.^60,61^ Time-explicit impact assessment methods, finally, remain an emerging field and are currently limited to climate change.^26,62^

Because a time-explicit LCO does not assess a fixed product system but decides which capacities to install and how to operate them, it requires further information that conventional LCI datasets do not carry. The flows of each process must be classified as installation- or operation-dependent, a distinction that traditional LCA datasets do not capture because traditional LCA methods can only assess a fixed product system. Each process additionally needs a technical lifetime *L*_p_, which bounds the operation of its vintages and, together with the rTDs, positions its end-of-life flows in time, while already existing installations enter as brownfield capacities with their own vintage and remaining lifetime. Temporal evolution must be specified for the foreground processes themselves and not only for the background, for which technology learning curves provide a starting point,^63^ although consistently and holistically capturing their environmental effects remains challenging.^64^ Beyond the process level, the demand has to be projected over the entire planning horizon, and stock- and flow-related limits require time-specific data on resource availability, such as regional water budgets or the primary supply of critical minerals. Most of these inputs are routine in process- or sector-specific models such as energy system optimization and can be adopted from there, so the practical bottleneck of time-explicit LCO lies less in the optimization data than in the temporal LCI data. Rather than requiring complete temporal coverage, optimex defaults to static data wherever temporal information is missing, so that temporal detail can be added incrementally as data become available.

Time-explicit LCO also increases computational requirements compared to static approaches, since the decision variables are indexed by both vintage and system time and the number of constraints grows accordingly. For the presented case study, the constrained evolution case comprises 3200 variables and 4501 constraints in total, which Gurobi^57^ solves to global optimality in 0.04 s on an Apple M2 Pro chip. These numbers remain modest because the background system is resolved into the intensity matrices ${\mathrm{\Lambda ̂}}_{t}$ before the optimization (Section 2.2.4), so that each background inventory enters the constraints and the objective as fixed parameters per impact category and time step, multiplied with the corresponding foreground flow. Constructing the intensity matrices dominates the overall computation time with 8 min on the same hardware, but has to be performed only once for a set of background systems, and can be re-used to assess different demands or constraint configurations.

Finally, the case study in Section 3.2 is chosen to illustrate the capabilities of the proposed time-explicit LCO framework rather than the methanol and pig iron sector, as it covers a relatively small set of processes, a single background scenario, and a demand that stays constant over the transition period. The two mechanisms revealed by the results, however, follow from the structure of the problem rather than from these specific data: overcapacities with preferential operation of newer vintages emerge whenever operational impacts dominate embodied impacts and process efficiencies are projected to improve significantly, which holds, for example, for many electrified processes drawing on increasingly decarbonizing electricity mixes. This strategy is only available because operation is bounded by, rather than equal to, the installed capacity (eq 11), so that vintages can be run at part load or idled entirely. Diversification emerges whenever a stock- or flow-related limit binds and alternative processes with complementary resource profiles exist; without such alternatives, the same limits instead shift installations in time or render the demand infeasible. The magnitude of both effects remains case-specific and depends on the assumed lifetimes, evolution trajectories, and limits. The choice of background scenario adds a further layer. For practical decision making, a range of scenarios should therefore be considered to understand how the identified pathways depend on the assumed trajectory.^65^ The framework is agnostic to the source of these scenarios and can equally operate on scenarios from alternative modeling paradigms or manually constructed projections, provided they are translated into time-specific LCI data.

### 4.3 Extensions and Future Directions

The framework’s mathematical structure lends itself to several extensions, spanning economic objectives, circular economy modeling, and advanced optimization techniques.

In practice, most real-world transition pathway optimization is driven by economics rather than environmental objectives alone. Adapting the proposed framework to cost minimization is straightforward: the environmental objective in eq 20a can be replaced by a cost objective (cf. Kätelhön et al.^16^), while time-explicit environmental targets are enforced as constraints. Technology-specific cost parameters, including capital expenditures, operational costs, and feedstock prices, can be incorporated as time-dependent coefficients, analogous to the vintage-dependent scaling factors (eq 10). The model can then reflect projected cost reductions from learning curves, evolving energy prices, or shifting raw material costs.

Beyond general cost parameters, the ability to resolve exact emission timing has specific implications for carbon markets, where emissions are typically priced using static metrics like GWP100, irrespective of when they occur. Yet, as carbon prices are projected to rise, emission costs become time-dependent. Time-explicit LCO could integrate such price trajectories directly, enabling optimization that minimizes financial risk alongside environmental impact. This integration is particularly relevant for sectors with long-lived infrastructure, where decisions to delay construction or prioritize early operational savings yield vastly different economic outcomes.

Alongside economic extensions, the framework provides a foundation for modeling circular economy dynamics. Internal material circularity in the foreground system can already be represented: secondary materials from end-of-life activities of earlier vintages enter product balances at specific system times, becoming available for subsequent installation phases. This representation, for example, enables pathways that optimize recycling loops within primary resource extraction limits. Such internal modeling could be complemented by interfacing with dynamic material flow analysis,^66^ allowing stocks and flows external to the system boundary to be accounted for and providing more comprehensive constraints on resource availability.^37,67^

Finally, by formulating time-explicit LCO as a mathematical optimization problem, the framework opens access to a broad range of established optimization methods. Future work can leverage multi-objective optimization to reveal trade-offs, for example between different environmental impact categories, or between different sustainability dimensions, from the economic objectives described above to social objectives, as demonstrated by Hülagü et al.^33^ Beyond the objective function, near-optimal methods can identify diverse pathways that offer decision-makers flexibility,^68^ while stochastic programming can ensure robustness against uncertainty,^16^ and mixed-integer^33^ and nonlinear^30^ formulations capture discrete investments and economies of scale, collectively refining time-explicit LCO as a tool for strategic planning of sustainable transitions.
